# Development of an Automated Liquid Biopsy Assay for Methylated Markers in Advanced Breast Cancer

**DOI:** 10.1158/2767-9764.CRC-22-0133

**Published:** 2022-06-01

**Authors:** Mary Jo Fackler, Suzana Tulac, Neesha Venkatesan, Adam J. Aslam, Timothy N. de Guzman, Claudia Mercado-Rodriguez, Leslie M. Cope, Bradley M. Downs, Abdul Hussain Vali, Wanjun Ding, Jennifer Lehman, Rita Denbow, Jeffrey Reynolds, Morgan E. Buckley, Kala Visvanathan, Christopher B. Umbricht, Antonio C. Wolff, Vered Stearns, Michael Bates, Edwin W. Lai, Saraswati Sukumar

**Affiliations:** 1 Department of Oncology, Johns Hopkins University School of Medicine, Baltimore, Maryland.; 2 Cepheid, Sunnyvale, California.; 3 Department of Oncology, Renmin Hospital of Wuhan University, Wuhan, Hubei, P.R. China.; 4 Department of Epidemiology, Johns Hopkins Bloomberg School of Public Health, Baltimore, Maryland.; 5 Department of Surgery, Johns Hopkins University School of Medicine, Baltimore, Maryland.

## Abstract

Current molecular liquid biopsy assays to detect recurrence or monitor response to treatment require sophisticated technology, highly trained personnel, and a turnaround time of weeks. We describe the development and technical validation of an automated Liquid Biopsy for Breast Cancer Methylation (LBx-BCM) prototype, a DNA methylation detection cartridge assay that is simple to perform and quantitatively detects nine methylated markers within 4.5 hours. LBx-BCM demonstrated high interassay reproducibility when analyzing exogenous methylated DNA (75–300 DNA copies) spiked into plasma (coefficient of variation, CV = 7.1%–10.9%) and serum (CV = 19.1%–36.1%). It also demonstrated high interuser reproducibility (Spearman *r* = 0.887, *P* < 0.0001) when samples of metastatic breast cancer (MBC, *N* = 11) and normal control (*N* = 4) were evaluated independently by two users. Analyses of interplatform reproducibility indicated very high concordance between LBx-BCM and the reference assay, cMethDNA, among 66 paired plasma samples [MBC *N* = 40, controls *N* = 26; Spearman *r* = 0.891; 95% confidence interval (CI) = 0.825–0.933, *P* < 0.0001]. LBx-BCM achieved a ROC AUC = 0.909 (95% CI = 0.836–0.982), 83% sensitivity and 92% specificity; cMethDNA achieved a ROC AUC = 0.896 (95% CI = 0.817–0.974), 83% sensitivity and 92% specificity in test set samples. The automated LBx-BCM cartridge prototype is fast, with performance levels equivalent to the highly sensitive, manual cMethDNA method. Future prospective clinical studies will evaluate LBx-BCM detection sensitivity and its ability to monitor therapeutic response during treatment for advanced breast cancer.

**Significance:**

We technically validated an automated, cartridge-based, liquid biopsy prototype assay, to quantitatively measure breast cancer methylation in serum or plasma of patients with MBC, that demonstrated high sensitivity and specificity.

## Introduction

Breast cancer is now the most common type of cancer worldwide (1). In newly updated data, Globocan 2020 estimates that there were nearly 2.3 million new breast cancer cases detected worldwide, with 685,000 deaths occurring due to metastatic breast cancer (MBC; ref. 1). In underdeveloped regions, most breast cancer is first detected as metastatic disease because patients remain asymptomatic for long periods of time before showing clinical manifestations (2). To increase survival and reduce morbidity and breast cancer–related deaths, clinicians need sensitive techniques to detect cancer, monitor therapeutic response, and recognize disease progression.

In recent years, there has been a shift toward evaluating liquid biopsy methods to detect cancer and monitor breast cancer progression in circulating tumor DNA (ctDNA) of patients with advanced disease (3–6). While not yet standard of care, these approaches have enabled clinicians to use tests for ctDNA in plasma or serum as a less invasive indicator of the presence of disease. A simple, noninvasive, liquid biopsy test would potentially allow clinicians to monitor disease burden and response to therapy more closely, enabling changes in treatment regimens that provide the highest probability of success, thereby using imaging modalities more cost-effectively.

Previously we developed cMethDNA, a highly sensitive and specific liquid biopsy laboratory assay based on multiplex, nested, real-time PCR to identify cumulative methylation (CM) levels of a panel of markers (7, 8). In patients with MBC, a 10-gene panel consisting of *AKR1B1*, *COL6A2*, *HOXB4*, *RASGRF2*, *RASSF1*, *HIST1H3C*, *GPX7*, *ARHGEF7*, *TMEFF2,* and *TM6SF1* detected ctDNA in 300 μL sera with high sensitivity (91%) and specificity (96%; ref. 7). In the Translational Breast Cancer Research Consortium (TBCRC005) cohort of patients undergoing chemotherapy for MBC, the index of CM (CMI) of a minimal 6-gene subset (*AKR1B1*, *HOXB4*, *RASGRF2*, *RASSF1*, *HIST1H3C*, and *TM6SF1*) was a strong predictor of survival outcomes in MBC (8). Yet, because cMethDNA involves a minimum of 1 week to complete and requires high technical competency, translation into a widely available diagnostic laboratory assay would be very challenging.

In fact, to date there is no commercially available *in vitro* diagnostic (IVD) circulating cell-free DNA (cfDNA) methylation assay developed for breast cancer (9–11). Therefore, our long-term goal is to use the principles of cMethDNA to develop an assay for routine use as a clinical management tool by making it faster and easier to perform through automation. As a first step, in collaboration with the diagnostics company Cepheid, and using their GeneXpert® platform (refs. 12–15; https://www.cepheid.com/) we have developed a prototype assay. The cartridge-based, Liquid Biopsy for Breast Cancer Methylation (LBx-BCM) assay is intended to be used at point of care as a rapid ancillary assay to support current clinical approaches to evaluate breast cancer. LBx-BCM is a prototype in development and is not for use in clinical diagnostic procedures and not reviewed by any regulatory body. Here, we report technical validation of LBx-BCM, demonstrating that it is possible to automate processing of plasma and serum samples and quantitatively assess DNA methylation for nine target genes within 4.5 hours, with less than 15 minutes of hands-on time.

## Materials and Methods

### Study Design and Sample Collections

We used prospective blood collections from studies that followed women with metastatic breast carcinoma at Johns Hopkins (JH): (i) A training set obtained from the JH Breast Program Repository (J0888, NCT01937039, collected from March 2015 to December 2015), (ii) a test set obtained from patients with MBC enrolled in the IMAGE II study (Individualized Molecular Analyses Guide Efforts in Breast Cancer, J16146, NCT02965755) and control normal or benign samples from J0888 collected from 2016 to 2020 and, (iii) two longitudinal studies (J0214, NCT00080665 and J0425, NCT00274768), collected at JH from 2004–2008. All J0888 samples used in training and test studies were from different donors.

### Ethics Approval and Consent to Participate

We obtained written informed consent from the patients following approval of each study from the JH Institutional Review Board. The studies were conducted in accordance with recognized ethical guidelines (Belmont Report).

### DNA Marker Selection

To ensure good breast cancer coverage for the LBx-BCM prototype assay, we chose nine CpG DNA markers from among a larger panel of cMethDNA genes that, together, recognized all four histologic subtypes of breast cancer (7, 8, 14, 16).

### The Prototype LBx-BCM

The GeneXpert® system (Cepheid) is a closed, automated PCR-based molecular diagnostic testing platform using self-contained cartridges to perform nucleic acid extraction and PCR. The LBx-BCM prototype was developed to meet the increased technical sensitivity required for detection of picograms of free ctDNA in blood. One cartridge is used for bisulfite conversion of unmethylated cytosine residues to uracil, which changes the DNA sequence specifically for unmethylated DNA, but not for methylated DNA (the conversion cartridge). Two additional cartridges are used for the performance of methylation-specific PCR (the methylation detection cartridges); these two detection cartridges contain reagents, in each cartridge, for nested multiplex real-time quantitative PCR of 4–5 target genes and ACTB as the internal reference, using six different fluorophores. Primer and probe sequences are presented in Supplementary Table S1. The entire assay is completed within 4.5 hours and requires approximately 15 minutes of hands-on time. LBx-BCM is a research use only prototype in development, not for use in diagnostic procedures, and has not been reviewed by any regulatory body.

### LBx-BCM Algorithm for Methylation

The method of calculating CM is described in Supplementary Table S2. Step 1: GeneXpert® software assigns the *C*_t_ at the end of the run; the user assigns *C*_t_ = 45 if no signals were detectable during the run; Δ*C*_t_ (*C*_t_ gene − *C*_t_ ACTB) is calculated to normalize all results to the ACTB reference DNA. If some samples have negative Δ*C*_t_ (*C*_t_ gene − *C*_t_ ACTB) for a gene, all samples are transformed by adding a constant value to give positive integers for that gene. Step 2: If Δ*C*_t_ (*C*_t_ gene − *C*_t_ ACTB) is higher than the historical replicate median of 300 copies + 13 Δ*C*_t_ units, the user adjusts to Δ*C*_t_ (*C*_t_ gene − *C*_t_ ACTB) = 0, thereby removing signals from the analysis that are too low to quantitate (less than 0.04 copies of target; Supplementary Fig. S1; Supplementary Table S2). Step 3: Gene methylation (M) = [1/Δ*C*_t_ (*C*_t_ gene − *C*_t_ ACTB)] * 1,200. This is a robust transformation intended to raise the methylation values from baseline and increase the assay dynamic range. Step 4: Calculate CM as follows, where CM = sum of M in the 9-gene panel.

### Sample Processing

Plasma from STRECK Cell-free DNA BCT tubes (STRECK, Omaha, NE, #218962) was collected, harvested, and frozen at −80°C within 5 days. Two sequential centrifugation steps ensured that the plasma was free of cells prior to freezing. Serum was harvested from serum separation tubes (BD, #367988), and frozen at −80°C within 4 hours. Plasma and serum were stored frozen at −80°C in aliquots. Before using, the samples were thawed at room temperature, inverted 10 times, then microcentrifuged at 14,000 rpm for 15 minutes at room temperature.

### The LBx-BCM Assay

For the LBx-BCM assay, plasma or serum (1.0 mL) was mixed with 50 μL proteinase K (600 units/mL; PK; Roche Diagnostics Corp.), 2.0 mL Lysis Buffer (Cepheid) and incubated for 10 minutes at room temperature. After incubation, absolute ethanol (1.5 mL) was added and the sample was loaded into the bisulfite conversion cartridge for processing (2.5 hours). The bisulfite-converted DNA sample was divided equally into two LBx-BCM methylation detection cartridges and methylation specific-PCR was performed (1 hour and 45 minutes; *AKR1B1*, *TM6SF1*, *ZNF671*, *TMEFF2* target genes and *ACTB* reference gene in Cartridge A; *COL6A2*, *HIST1H3C*, *RASGRF2*, *HOXB4*, *RASSF1* target genes and ACTB in Cartridge B). The following reactions were run in each methylation detection cartridge: (i) a methylation-independent, nested multiplexed PCR that preamplified the 9-gene panel for 20 cycles, and (ii) a methylation-specific, nested quantitative 6-plex real-time PCR that uses internal primers and 6 fluorophores (one per marker) to quantitate amplicons generated in the first PCR. The assay is completed within 4.5 hours including approximately 15 minutes of preparation time.

### Preparation of Analytic Replicates

We spiked 600, 300, 150, or 0 copies of a laboratory stock of methylated human control DNA (#N2131, Promega Corp.) that was previously quantified by digital droplet PCR into 1.0 mL of commercial normal plasma or serum (female human pooled plasma, K_2_EDTA anticoagulated or pooled serum, BioIVT). After adding PK (50 μL), lysis buffer (2 mL) and absolute ethanol (1.5 mL), each sample was transferred to a bisulfite cartridge. Within this cartridge, DNA was extracted, then converted with sodium bisulfite (D5030-1, Lightning Conversion Reagent, Zymo Research) and afterward transferred in equal amounts to each of two detection cartridges for quantitative nested methylation-specific real-time PCR.

### Interuser Reproducibility

J0888 repository samples obtained from patients with MBC (*N* = 11) and normal controls (*N* = 4) were aliquoted into duplicate sample sets. One set was tested by User A and the other set was tested by User B on separate days using the same reagents. Users were blinded to the origin of the samples. LBx-BCM was performed as described above. Interuser concordance was evaluated using the Spearman correlation coefficient.

### Statistical Analysis

Analyses of independent groups were performed and data were visualized using box whisker plots. Differences between groups were evaluated using the nonparametric Mann–Whitney test. The performance of the 9-gene panel was characterized by estimating the area under the receiver operating characteristic curve (AUC), sensitivity, specificity, and likelihood ratio along with the 95% confidence intervals (CI). Classification accuracy = TP + TN/TP + TN + FP + FN using ROC-derived laboratory methylation cutoffs (38.5 CM units for LBx-BCM; 1.5 CM units for cMethDNA). All statistical tests were two sided and considered statistically significant at *P* < 0.05. Spearman correlation was performed to compare the CM of the reference laboratory assay, cMethDNA, with CM obtained in the LBx-BCM system in the test set samples (40 cancer and 26 control noncancer samples). GraphPad Prism version 9.0 (GraphPad Software) was used for all analyses.

### Data Availability

Patient datasets generated and/or analyzed during this study are not publicly available due to the sensitivity of the data, but are available from the corresponding author upon reasonable request.

## Results

### The LBx-BCM Prototype Assay

**FIGURE 1:**
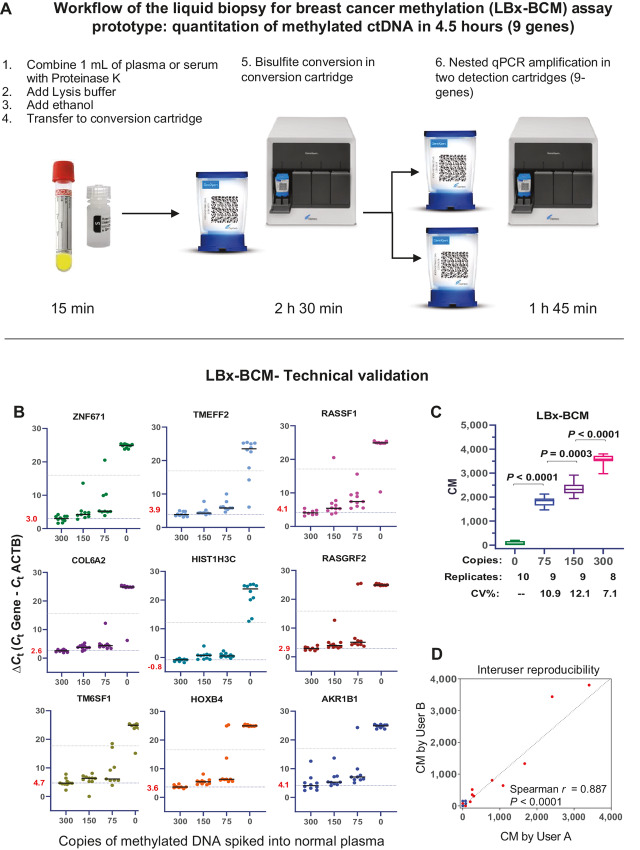
LBx-BCM and its technical validation. **A,** Workflow of the LBx-BCM assay**. B,** Analytic sensitivity of LBx-BCM for each gene in the 9-gene panel relative to ACTB. We spiked 300, 150, 75, and 0 copies of fully methylated DNA into 0.5 mL aliquots of pooled commercial normal plasma. Replicate LBx-BCM assays were performed. The ∆*C*_t_ (*C*_t_ Gene − *C*_t_ ACTB) of samples is plotted on the *y-*axis for each gene. Number of copies of methylated DNA spiked into plasma is indicated on the *x-*axis. For each gene, the median ∆*C*_t_ (*C*_t_ Gene − *C*_t_ ACTB) for 300 copies of spiked DNA is indicated numerically in red to the left of the *y-*axis and by the bottom dotted line. The top dotted line indicates median ∆*C*_t_ for 300 copies + 13 for each marker. **C,** CM. The CM of 9 genes was calculated for each run of 300, 150, 75, and 0 copies as described in the Materials and Methods. Box plots represent the median and range of CM of the 9-gene panel among spiked-in replicates. Mann–Whitney analysis was performed to determine whether methylation was significantly different as indicated by *P* values. The CV, expressed as percent (CV %) is indicated. For the calculation of gene methylation (M) and CM, we utilized the replicate median Δ*C*_t300 DNA copies_ indicated in Supplementary Table S2. Methylated DNA was spiked into serum for training and longitudinal sets and spiked into plasma for the test set. **D,** Interuser reproducibility. To test reproducibility between users, a total of 15 plasma samples including 11 from patients with MBC, and four from healthy controls were aliquoted in duplicate and each set was assayed for LBx-BCM by two users on different days. Data from user A (*x*-axis) and user B (*y*-axis) are plotted (Spearman *r* = 0.887, *P* < 0.0001).

The LBx-BCM's quantitative PCR workflow is depicted in Fig. 1A. Steps 1–4 involve pre-processing of the sample for DNA extraction. In step 5, the mixture is placed in the bisulfite conversion cartridge. In step 6, the bisulfite-treated DNA is divided equally into two LBx-BCM methylation detection cartridges to amplify and detect nine methylated genes (up to five methylated genes plus ACTB per cartridge). At the end of the assay (4.5 hours), the cycle threshold (*C*_t_) for each gene is provided.

For LBx-BCM marker development, we selected a 9-marker panel. We selected primer/probe combinations that performed optimally in the presence of the other markers and fluorophores in the 6-plex reaction in each cartridge (7, 8, 14). The final 9-gene panel consisted of *HOXB4, RASGRF2, AKR1B1, TM6SF1, COL6A2, HIST1H3C, TMEFF2, RASSF1,* and *ZNF671*. Primer and probe sequences for the gene panel are shown in Supplementary Table S1.

### Evaluation of the Analytic Performance of the LBx-BCM Prototype

#### Interassay Reproducibility

The challenge in development of an automated assay for ctDNA is the ability to detect only a few hundred picograms or less of target DNA in a vast abundance of normal cfDNA. We developed the cartridge-based LBx-BCM assay (Fig. 1A), including the method for calculating CM (Supplementary Fig. S1; Supplementary Table S2). The analytic performance of the assay was evaluated by spiking replicates of 300, 150, 75, and 0 copies (1 ng–250 pg) of fully methylated target DNA into 0.5 mL of either commercial pooled normal plasma (Fig. 1B and C; Supplementary Table S3) or normal serum (Supplementary Fig. S2A and S2B; Supplementary Table S3). On the basis of the Δ*C*_t_ (*C*_t_ Gene − *C*_t_ ACTB), scatter diagrams showed that nearly all replicates of 75–300 copies of target DNA were detected. In the absence of target DNA (0 spiked copies), either the input DNA was too low to quantitate, or no PCR amplification was observed (Fig. 1B; Supplementary Fig. S1; Supplementary Table S3). The Δ*C*_t_ increased with decreasing number of copies of target for each gene. CM of the 9-gene panel was significantly different for 0 versus 75 copies (*P* < 0.0001), 75 versus 150 copies (*P* = 0.0003), and 150 versus 300 copies (*P* < 0.0001; Mann–Whitney Analysis; Fig. 1C) in spiked normal plasma. Similar results were observed in spiked normal serum (Supplementary Fig S2A; Mann–Whitney analysis; Supplementary Fig. S2B). For calculation of gene methylation (M) and CM of all genes (Supplementary Table S2), we used the replicate control median Δ*C*_t_ of 300 copies, as shown in Supplementary Table S3.

#### Interuser Reproducibility

We evaluated LBx-BCM reproducibility between users to determine whether the method gave similar results independent of the operator. A total of 15 samples, including patients with MBC (*N* = 11), and healthy controls (*N* = 4), were divided into duplicate sets and assayed on different days using cartridges from the same batch. The Spearman *r* = 0.887 indicated a high level of interuser reproducibility (Fig. 1D).

#### LBx-BCM–Based Detection of MBC in the Training Set

The LBx-BCM ctDNA method was initially evaluated in JH repository J0888 samples (patient characteristics and sample sets presented in Tables 1 and 2) to verify that LBx-BCM could distinguish between MBC versus normal serum using circulating cfDNA. For many of these patients, blood was collected while they were undergoing chemotherapy. We examined CM of the 9-marker panel in serum samples (MBC, *N* = 20; control normal, *N* = 20), and observed significantly higher methylation in the cancer sera compared with normal controls as shown in the histogram (Supplementary Fig. S3A) and in box whiskers plot (Supplementary Fig. S3B; Mann–Whitney test *P* = 0.002). The ROC-derived threshold that provided the highest combined sensitivity and specificity was 38.5 CM units (Supplementary Fig. S3C). At this threshold the ROC AUC = 0.766 (95% CI, 0.616–0.916; *P* = 0.004), with 75% sensitivity (95% CI, 53.1–88.8) and 65% specificity (95% CI, 43.3–81.9).

**TABLE 1:** Clinical characteristics of cases and controls in the study

Characteristics	J0888	J16146 (IMAGE II)—J0888	J0214—J0425
**A. Metastatic breast cancer**	**Training set**	**Test set**	**Longitudinal set**
Patient characteristics	*n* = 20	*n* = 40	*n* = 22
Race			
Caucasian	17	25	16
Black	1	12	5
Other	2	3	1
Location of disease			
Visceral	0	4	3
Nonvisceral	8	5	1
Both	12	28	18
Unknown	0	3	0
Receptor status			
ER/PR-positive, HER2-negative	17	29	12
ER/PR-positive, HER2-unknown	0	1	0
HER2-positive	2	1	4
Triple-negative (ER,PR,HER2 negative)	1	8	6
Unknown	0	1	0
Received prior chemotherapy for MBC	8	33	9
No. of prior treatment regimens (all, incl. hormone)			
0	1	1	13
1	6	8	2
2	2	9	5
3	3	9	1
≥ 4	8	13	1
Age			
Median	58	59	54
Range	29–82	27–80	28–73
**B. Benign/Normal Controls**	**Training set**	**Test set**	**Longitudinal set**
Patient characteristics	*n* = 20	*n* = 26	*n* = 0
Race			
Caucasian	10	16	
Black	10	9	
Other	0	1	
Age			
Median	58	53	

**TABLE 2:** Study design and sample sets used for evaluating performance of LBx-BCM

Sample sets					
A. Performance in training and test sets					
	Blood				
	Serum		Plasma		
Sample sets	Cancer	Normal	Cancer	Benign	Normal
Training set for LBX-BCM—J0888 Metastatic Breast Cancer, Healthy Controls, Supplementary Fig. S3	20	20	0	0	0
Test set—for LBx-BCM and cMethDNA—IMAGE II Trial Metastatic Breast Cancer and J0888 Benign and Healthy Controls, Figs. 2 and 3	0	0	40	17	9
**B. Changes in methylation during chemotherapy-longitudinal study**					
	**Serum, Baseline + follow-up**				
**Sample sets, Figs. 4 and Supplementary Fig. S5**	**Patients**		**Total samples**		
J0425 Metastatic Breast Cancer	13		46		
J0214 Metastatic Breast Cancer	9		28		
IMAGE II—Individualized Molecular Analyses Guide Efforts in Breast Cancer, J16146					
All normal samples are from different individuals					

#### Accuracy of LBx-BCM to Detect MBC in the Test Set

**FIGURE 2:**
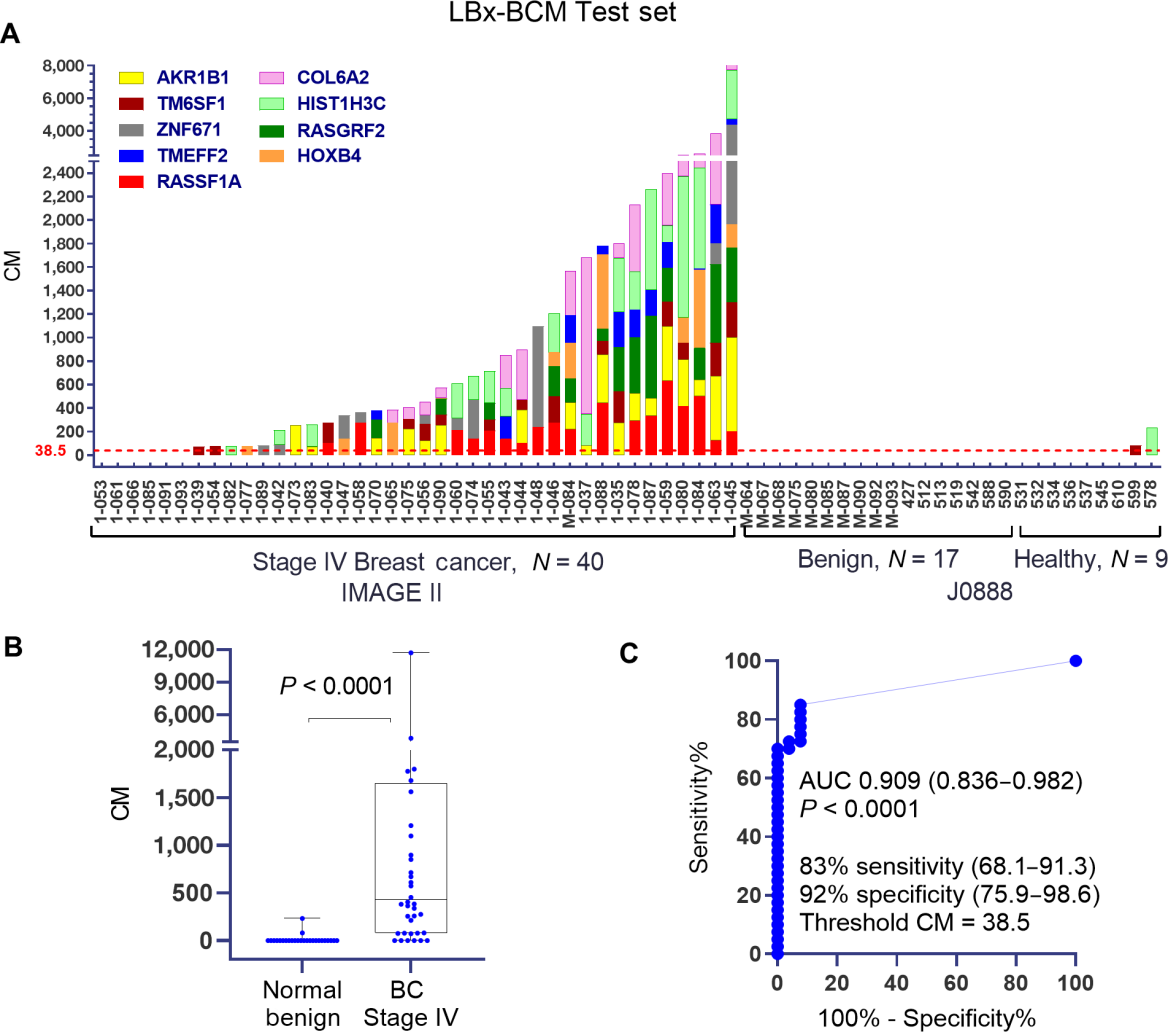
Performance of LBx-BCM in test set of IMAGE II/J0888 study samples. **A,** Histograms indicate the magnitude of CM (*y*-axis) in each plasma sample (*x*-axis). The height of each colored segment indicates the extent of methylation for each individual gene. **B,** A box plot of CMs shows significant differences in ctDNA methylation between cancer and benign/normal samples (*P* < 0.0001, Mann–Whitney). **C,** Detection sensitivity and specificity. The ROC analysis indicated LBx-BCM had 83% sensitivity and 92% specificity to detect cancer with an AUC of 0.909. The ROC analysis utilized the 38.5 CM unit cutoff established in training set samples (Supplementary Fig. S3).

We then locked existing assay parameters and tested an independent, well annotated and prospectively collected set of plasma samples. The cancer samples were from the IMAGE II trial [MBC *N* = 40, and controls from the J0888 repository (benign breast disease, *N* = 17; healthy normal, *N* = 9)]. Patient characteristics and sample sets are shown in Tables 1 and 2. Consistent with the results in the training set, LBx-BCM detected significantly more methylation in plasma samples from breast cancer than in normal or benign samples as shown in the histogram (Fig. 2A) and box whiskers plots (Fig. 2B; Mann–Whitney test *P* < 0.0001). At the training set CM threshold of 38.5 units (Supplementary Fig. S3C), the test set ROC AUC = 0.909 (95% CI = 0.836–0.982, *P* < 0.0001), with a sensitivity of 83% (95% CI = 68.1–91.3) and a specificity of 92% (95% CI = 75.9–98.6; Fig. 2C). The endogenous reference gene ACTB Ct in the test set for stage IV samples ranged from 16.0 to 27.8, and in the normal samples ranged from 21.0 to 27.4; the difference between cancer and normal was statistically significant (Supplementary Fig. S4; Mann–Whitney *P* < 0.0001).

#### Interplatform Concordance between LBx-BCM and cMethDNA

**FIGURE 3:**
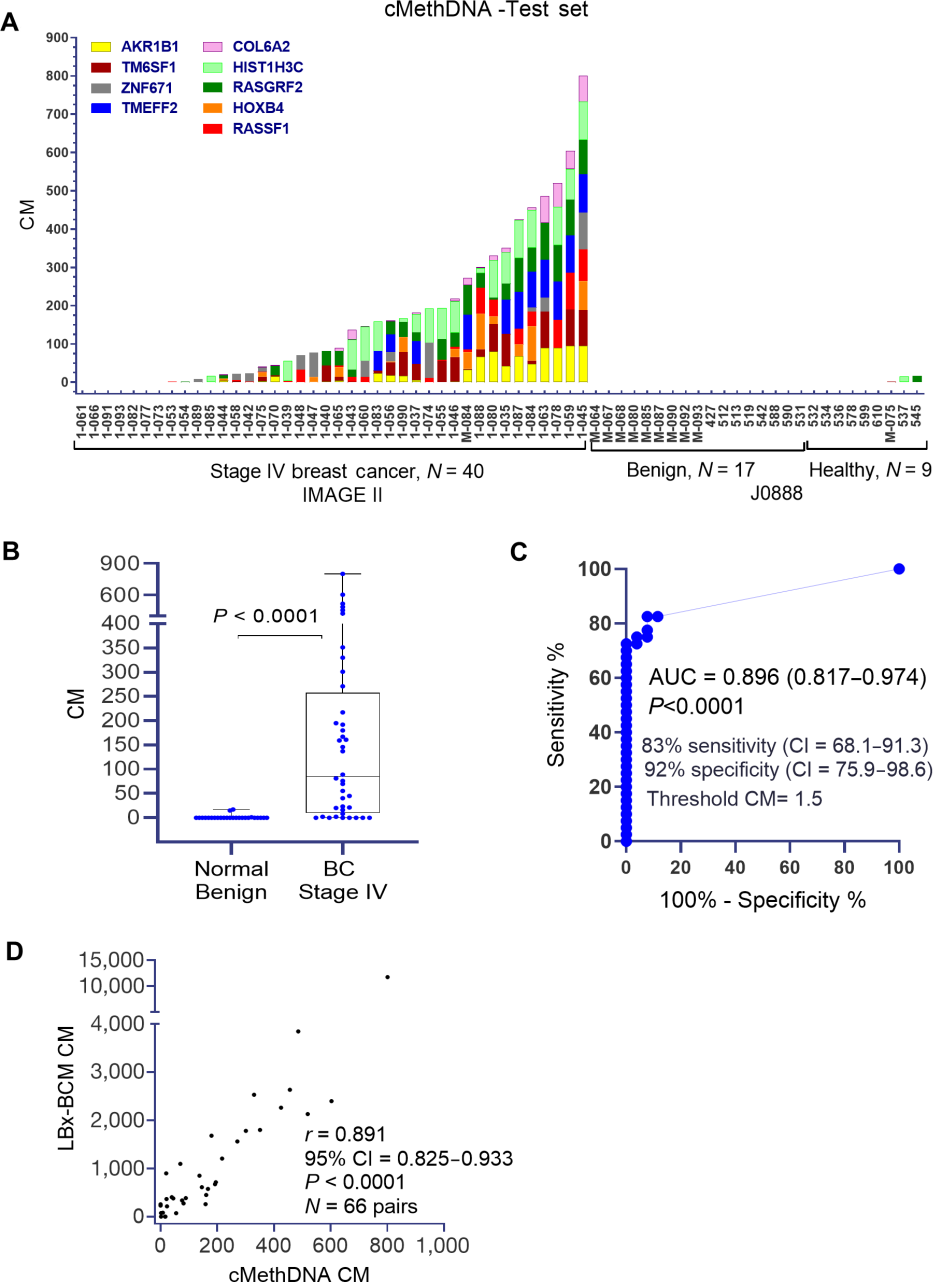
Assay concordance between LBx-BCM and cMethDNA. We also tested the IMAGE II/J0888 test set samples using the reference cMethDNA assay to directly compare the methylation measures by both platforms when tested on the same samples. **A,** Histogram analysis. cMethDNA histogram indicates the magnitude of CM (*y*-axis) for each sample (*x*-axis). The height of each colored segment indicates the extent of methylation for individual genes. **B,** Box plot shows CM in samples of normal/benign versus cancer ctDNA methylation (*P* < 0.0001, Mann–Whitney). **C,** Detection sensitivity and specificity. ROC analysis indicated cMethDNA had 83% sensitivity and 92% specificity to detect cancer using a cutoff of 1.5 CM units. **D,** Interplatform assay concordance. CM was plotted for LBx-BCM (*y*-axis) and cMethDNA (*x*-axis) for individual samples. The Spearman *r* = 0.891 indicated high level of concordance between these two platforms.

LBx-BCM and cMethDNA assays utilize nearly identical primer/probe sequences and similar nested quantitative multiplex methylation-specific PCR strategies. However, cMethDNA normalizes methylation to a gene-specific recombinant standard of 50 methylated copies spiked into 300 μL of plasma or serum prior to purification of DNA, while LBx-BCM normalizes methylation to the endogenous actin reference in the DNA present in 500 μL plasma or serum. As a technical verification step to determine whether LBx-BCM achieved a similar level of performance as cMethDNA, we performed cMethDNA on the entire IMAGE II/J0888 test set (Fig. 3). Consistent with the LBx-BCM results, cMethDNA detected significantly more methylation in plasma samples from breast cancer than from normal or benign individuals as shown in histogram and box whiskers plots (Fig. 3A and B; Mann–Whitney test *P* < 0.0001). At the CMI threshold of 1.5 units, for cMethDNA the ROC AUC = 0.896 (95% CI = 0.817–0.974; *P* < 0.001), with a sensitivity of 83% (95% CI = 68.1–91.3) and a specificity of 92% (95% CI = 75.9 –98.6; Fig. 3C). LBx-BCM and cMethDNA methylation results were highly concordant (Spearman *r* = 0.891, *P* < 0.0001; *N* = 66 paired samples; Fig. 3D). Descriptive statistics for interplatform reproducibility between LBx-BCM and cMethDNA are provided in Table 3.

**TABLE 3:** Interplatform reproducibility between LBx-BCM and cMethDNA

A. Cumulative methylation (nine genes) in MBC and normal sera, test set samples^a^	LBx-BCM		cMethDNA^b^	
	Control	MBC	Control	MBC
*N*	26	40	26	40
Minimum	0	0	0	0
25% Percentile	0	79	0	10
Median	0	428	0	85
75% Percentile	0	1,651	0	258
Maximum	231	11,729	17	801
Mean	12	1,117	1	166
Lower 95% CI of mean	0	490	0	103
Upper 95% CI of mean	31	1,744	3	229
**B. ROC analysis**	**LBx-BCM**		**cMethDNA**	
Area under the curve	0.909		0.896	
95% confidence interval	0.836 to 0.982		0.817 to 0.974	
*P*	< 0.0001		< 0.0001	
Sensitivity	83%		83%	
Specificity	92%		92%	
ROC CM threshold^c^	> 38.5		> 1.5	
Likelihood ratio	11.1		10.7	
Classification accuracy	89%		85%	

^a^The test set samples from the J16146 (IMAGE II)/J0888 studies were used.

^b^cMethDNA was used as a reference assay.

^c^Positive for methylation is defined as ≥ the ROC CM threshold.

#### Changes in LBx-BCM Methylation During Treatment of MBC

**FIGURE 4:**
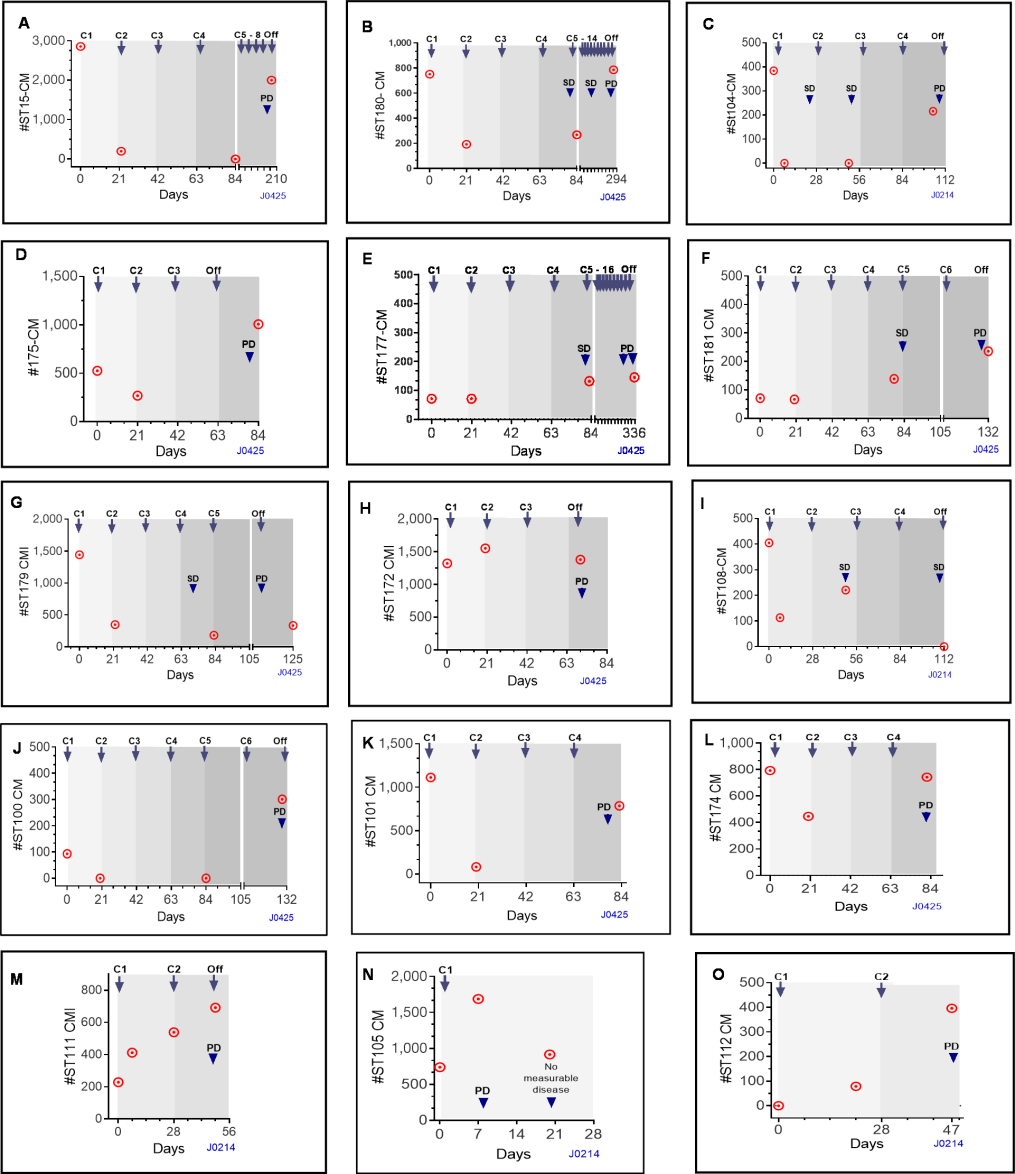
Changes in methylation in ctDNA by LBx-BCM in response to chemotherapy. Patients with MBC were enrolled in the J0214 and J0425 studies and received either 21-day cycles of capecitabine or 28 days of docetaxel, indicated by C1, C2, and so on, and the shaded background. **A–O,** Blood was collected from each patient at baseline, and on days indicated in the plots after the start of the new chemotherapy. CM measured by LBx-BCM in each patient sample is shown on the *x*-axis, indicated by dots. Additional diagrams are presented in Supplementary Fig. S5. PD, progressive disease; SD, stable disease.

We had previously reported results of longitudinal studies in serial blood collections for cMethDNA (7, 8). Because LBx-BCM demonstrated excellent concordance with this assay, we predicted that LBx-BCM methylation levels would also change during the course of chemotherapy. We analyzed CM by LBx-BCM in serum samples obtained from MBC patients in two prospective clinical studies conducted at JH—J0214 and J0425. Serum was collected prior to the initiation of treatment (baseline), 18–49 days (median 21 days) after starting a new line of treatment, and upon completion of additional cycles. Patients received either 28-day cycles of docetaxel or 21-day cycles of capecitabine. Representative plots of LBx-BCM methylation are shown in Fig. 4 and Supplementary Fig. S5. In these heavily pretreated patients with stage IV breast cancer, changes in CM occurred frequently during the course of treatment. For many patients, there was an initial reduction in methylation after the initiation of therapy. Increased methylation was observed among patients who progressed on treatment and among some patients with stable disease (Fig. 4; Supplementary Fig. S5).

## Discussion

Widespread diagnostic implementation of assays that detect ctDNA has not occurred. This is largely due to the technical complexities of such assays and the extensive time required to conduct them (9, 17–19). We sought to overcome these obstacles by developing, to the best of our knowledge, the first automated ctDNA methylation assay capable of simultaneously quantitating methylation levels in a panel of markers. Our goal was to develop an assay that would be sophisticated yet simple to perform in underserved regions worldwide, and could be used at point of care to provide same-day feedback to clinicians and patients (7, 8, 14, 20). We report the development of a nested, quantitative, multiplexed methylation-specific PCR assay, called LBx-BCM run on the GeneExpert® system. It can be performed in approximately 4.5 hours sample to answer and it uses many of the same markers and principles as the highly sensitive manual cMethDNA method that served as its foundation (7, 8, 14). LBx-BCM is a prototype for research use only.

In the current study, we report technical development and validation of LBx-BCM. We chose a ctDNA marker panel consisting of *HOXB4, RASGRF2, AKR1B1, TM6SF1, COL6A2, HIST1H3C, TMEFF2, RASSF1,* and *ZNF671* (7, 8, 14) and then developed LBx-BCM using the training set of J0888 repository samples. LBx-BCM was validated by performing interassay, interuser and interplatform reproducibility studies comparing LBx-BCM with the reference method cMethDNA (7). Interassay reproducibility studies demonstrated that LBx-BCM was able to detect statistically significant differences in CM between cartridges spiked with 75, 150, and 300 copies of methylated DNA (*P* < 0.0001 to *P* = 0.0003). Interuser reproducibility studies showed that the LBx-BCM assay run by two different users on different days performed consistently at a high level of concordance (*N* = 15; Spearman *r* = 0.887, *P* < 0.0001). Most importantly, with the test set of prospectively collected IMAGE II MBC trial samples, the interplatform reproducibility study demonstrated impressive overall concordance between LBx-BCM CM and cMethDNA CMI results run on the same samples (Spearman *r* = 0.891, *P* < 0.0001, *N =* 66 paired test set samples). ROC performance and diagnostic accuracy were nearly identical between LBx-BCM and cMethDNA. LBx-BCM achieved high sensitivity (83%) and specificity (92%) for an overall diagnostic accuracy of 89%, and a ROC AUC of 0.909. By comparison, for cMethDNA, the sensitivity was 83%, specificity was 92%, for an overall diagnostic accuracy of 85% and ROC AUC = 0.896.

Our automated LBx-BCM system has several important strengths. It is highly sensitive and specific, is technically simple and convenient, has a fast turnaround time, and performs with a high level of accuracy. The detection sensitivity of LBx-BCM is as good or better in advanced breast cancer as other reported quantitative methylation—specific PCR assays, reviewed in Constancio and colleagues (21). For example, Shan and colleagues (22) reported that six methylated markers could discriminate between breast cancer patients and healthy women with a sensitivity of 79.6% and a specificity of 72.4% (AUC, 0.727 (95% CI, 0.712–0.742), *P* < 0.001. Klein and colleagues reported that a multicancer early detection (MCED) blood test that uses methylated cfDNA sequencing combined with machine learning. The assay detected cancer signals and predicted its origin in multiple cancer types with high accuracy (23). Using large study sets, for cancer signal detection, an overall sensitivity of 51.5% (49.6%–53.3%) at a high specificity of 99.5% was achieved. Analyzing a relatively small set of stage IV breast cancers (*N* = 20), signal detection reached a sensitivity of 90.9% at the same high level of specificity (23), although the sensitivity to predict tumor origin was only 29.6%. Shen and colleagues (24) used 1–10 ng cfDNA to perform methyl-DNA immunoprecipitation followed by high-throughout sequencing to profile methylation patterns typical of tumor cfDNA in several tumor types. However, validation of the method based on differentially methylated regions was not performed in breast cancer (24).

A second strength of LBx-BCM is the minimal number of off-board steps. The short time requirement of only 4.5 hours is unique to this assay. Another strength is the fact that, unlike many other blood-based assays based on genomic sequencing (25–27), a sample of the primary or metastatic tumor is not required, resulting in substantial savings in time and cost. Taken together, these considerations suggest that our assay could be widely applied at the point of care. We also believe it can be easily adapted to a variety of cancer types.

However, the main limitation of our study is its small sample size. Also, in our studies we noted that LBx-BCM performed less well on serum in comparison with plasma. In reproducibility analyses of 75–300 copies of spiked exogenous methylated DNA, the coefficient of variation (CV) was tighter in plasma (CV = 7.1%–10.9%; Fig. 1D) than in serum (CV = 19.0%–36.1%; Supplementary Fig. S2). Consistent with these observations, plasma samples in the test set showed better sensitivity and specificity (83% and 92%, respectively) than serum in the training set (75% sensitivity and 65% specificity). However, this small training set of sera was from patients who were currently on treatment. We cannot definitively determine whether it was the quality of the sample or the assay itself that contributed to the lower performance in the training set. In addition, although our results are promising, our study cohort was primarily from patients (29/39) with ER^+^/PR^+^/HER2^−^ breast cancer. The LBx-BCM assay needs to be evaluated in large, prospectively designed studies which include a balanced representation of all histologic subtypes of breast cancer and a greater ethnic diversity. Such a cohort has already been identified in the prospective TBCRC 005 trial of patients with stage IV breast cancer for whom serial blood sampling was performed at baseline, 3–4 weeks after initiation of a new chemotherapy treatment and 8–12 weeks later (8). It would also be important to evaluate the performance of this automated system in detecting disease in patients with earlier stages of breast cancer.

In conclusion, we have developed and technically validated a quantitative multiplexed and automated assay for methylated markers in the GeneXpert® system for assaying cfDNA from a liquid biopsy which can be implemented at the point of care.

## Supplementary Material

Supplementary Fig S1 Figure shows the relationship between PCR Cycle threshold (Ct) and target DNA copy (0-300 copies) input

Supplementary Fig S2 Figure shows the analytical sensitivity of LBx-BCM based detection of fully methylated DNA (0-300 copies) spiked into normal serum separately for each gene in the panel in 10-11 replicate assays

Supplementary Fig S3 Figure shows the perfomance of LBx-BCM in the training set samples. A histogram shows cumulative methylation for each sample, and a box plot shows significnt difference of methylation in serum of metastatic breast cancer patients compared to normal individuals (Mann Whitney p= 0.002)

Supplementary Fig S4 Figure shows ACTB reference gene DNA levels plotted for each of the 132 study samples. Stage IV breast cancer patient sera had significantly higher total ACTB DNA (lower Ct) compared to normal (Mann Whitney P < 0.0001). Descriptive statistics are shown for normal vs. cancer and for methylation Cartridge A vs Cartridge B

Supplementary Fig S5 Figure shows changes in LBx-BCM methylation in response to chemotherapy in 7 additional patient longitudinal serial samples. LBx-BCM was performed and cumulative methylation (CM) (Y-axis) is plotted from serum samples drawn at baseline (0 days) and immediately before each treatment cycle (X-axis). For each patient, treatment cycles are shown as a shaded area. PD, progressive disease; SD, stable disease (SD).

Supplementary Table S1 Table contains sequences of primers and probes used to amplify the nine markers and ACTB in the LBx-BCM assay

Supplementary Table S2 The table provides a step by step guide to calculate methylation in each gene followed by cumulative methylation using the algorithm derived in this paper.

Supplementary Table S3 The table provides descriptive statistics and coefficient of variation for methylation in each gene in replicate LBx-BCM analyses of 300 copies of fully methylated DNA spiked into normal serum or plasma

## References

[1] Sung H , Ferlay J, Siegel RL, Laversanne M, Soerjomataram I, Jemal A, . Global cancer statistics 2020: GLOBOCAN estimates of incidence and mortality worldwide for 36 cancers in 185 countries. CA Cancer J Clin 2021;71:209–49. 33538338 10.3322/caac.21660

[2] Barrett T , Bowden DJ, Greenberg DC, Brown CH, Wishart GC, Britton PD. Radiological staging in breast cancer: which asymptomatic patients to image and how. Br J Cancer 2009;101:1522–8. 19861999 10.1038/sj.bjc.6605323 PMC2778507

[3] Alimirzaie S , Bagherzadeh M, Akbari MR. Liquid biopsy in breast cancer: a comprehensive review. Clin Genet 2019;95:643–60. 30671931 10.1111/cge.13514

[4] Zhang H , Pandey S, Travers M, Sun H, Morton G, Madzo J, . Targeting CDK9 reactivates epigenetically silenced genes in cancer. Cell 2018;175:1244–58. 30454645 10.1016/j.cell.2018.09.051 PMC6247954

[5] Matsutani A , Udagawa C, Matsunaga Y, Nakamura S, Zembutsu H. Liquid biopsy for the detection of clinical biomarkers in early breast cancer: new insights and challenges. Pharmacogenomics 2020;21:359–67. 32284011 10.2217/pgs-2019-0130

[6] Stastny I , Zubor P, Kajo K, Kubatka P, Golubnitschaja O, Dankova Z. Aberrantly methylated cfDNA in body fluids as a promising diagnostic tool for early detection of breast cancer. Clin Breast Cancer 2020;20:e711–22. 32792225 10.1016/j.clbc.2020.05.009

[7] Fackler MJ , Lopez Bujanda Z, Umbricht C, Teo WW, Cho S, Zhang Z, . Novel methylated biomarkers and a robust assay to detect circulating tumor DNA in metastatic breast cancer. Cancer Res 2014;74:2160–70. 24737128 10.1158/0008-5472.CAN-13-3392 PMC4327879

[8] Visvanathan K , Fackler MS, Zhang Z, Lopez-Bujanda ZA, Jeter SC, Sokoll LJ, . Monitoring of serum DNA methylation as an early independent marker of response and survival in metastatic breast cancer: TBCRC 005 prospective biomarker study. J Clin Oncol 2017;35:751–8. 27870562 10.1200/JCO.2015.66.2080 PMC5455421

[9] Taryma-Leśniak O , Sokolowska KE, Wojdacz TK. Current status of development of methylation biomarkers for *in vitro* diagnostic IVD applications. Clin Epigenetics 2020;12:100. 32631437 10.1186/s13148-020-00886-6 PMC7336678

[10] Beltrán-García J , Osca-Verdegal R, Mena-Mollá S, García-Giménez JL. Epigenetic IVD tests for personalized precision medicine in cancer. Front Genet 2019;10:621. 31316555 10.3389/fgene.2019.00621 PMC6611494

[11] Locke WJ , Guanzon D, Ma C, Liew YJ, Duesing KR, Fung KYC, . DNA methylation cancer biomarkers: translation to the clinic. Front Genet 2019;10:1150. 31803237 10.3389/fgene.2019.01150 PMC6870840

[12] García-Gutiérrez V , Gómez-Casares MT, Puerta JM, Alonso-Domínguez JM, Osorio S, Hernández-Boluda JC, . A BCR-ABL1 cutoff of 1.5% at 3 months, determined by the GeneXpert system, predicts an optimal response in patients with chronic myeloid leukemia. PLoS One 2017;12:e0173532. 28278193 10.1371/journal.pone.0173532 PMC5344481

[13] Wu NC , Wong W, Ho KE, Chu VC, Rizo A, Davenport S, . Comparison of central laboratory assessments of ER, PR, HER2, and Ki67 by IHC/FISH and the corresponding mRNAs (ESR1, PGR, ERBB2, and MKi67) by RT-qPCR on an automated, broadly deployed diagnostic platform. Breast Cancer Res Treat 2018;172:327–38. 30120700 10.1007/s10549-018-4889-5 PMC6208911

[14] Downs BM , Mercado-Rodriguez C, Cimino-Mathews A, Chen C, Yuan J-P, Van Den Berg E, . DNA methylation markers for breast cancer detection in the developing world. Clin Cancer Res 2019;25:6357–67. 31300453 10.1158/1078-0432.CCR-18-3277 PMC6825533

[15] Gupta S , Mccann L, Chan YGY, Lai EW, Wei W, Wong PF, . Closed system RT-qPCR as a potential companion diagnostic test for immunotherapy outcome in metastatic melanoma. J Immunother Cancer 2019;7:254. 31533832 10.1186/s40425-019-0731-9 PMC6751819

[16] Stearns V , Fackler MJ, Hafeez S, Bujanda ZL, Chatterton RT, Jacobs LK, . Gene methylation and cytological atypia in random fine-needle aspirates for assessment of breast cancer risk. Cancer Prev Res 2016;9:673–82. 10.1158/1940-6207.CAPR-15-0377 PMC4970896 27261491

[17] Colmenares R , Álvarez N, Barrio S, Martínez-López J, Ayala R. The minimal residual disease using liquid biopsies in hematological malignancies. Cancers 2022;14:1310. 35267616 10.3390/cancers14051310 PMC8909350

[18] Jagannathan G , White MJ, Xian RR, Emens LA, Cimino-Mathews A. A new landscape of testing and therapeutics in metastatic breast cancer. Surg Pathol Clin 2022;15:105–20. 35236627 10.1016/j.path.2021.11.007

[19] Heitzer E , Van Den Broek D, Denis MG, Hofman P, Hubank M, Mouliere F, . Recommendations for a practical implementation of circulating tumor DNA mutation testing in metastatic non-small-cell lung cancer. ESMO Open 2022;7:100399. 35202954 10.1016/j.esmoop.2022.100399 PMC8867049

[20] Stefansson OA , Moran S, Gomez A, Sayols S, Arribas-Jorba C, Sandoval J, . A DNA methylation-based definition of biologically distinct breast cancer subtypes. Mol Oncol 2015;9:555–68. 25468711 10.1016/j.molonc.2014.10.012 PMC5528700

[21] Constâncio V , Nunes SP, Henrique R, Jerónimo C. DNA methylation-based testing in liquid biopsies as detection and prognostic biomarkers for the four major cancer types. Cells 2020;9:624. 32150897 10.3390/cells9030624 PMC7140532

[22] Shan M , Yin H, Li J, Li X, Wang D, Su Y, . Detection of aberrant methylation of a six-gene panel in serum DNA for diagnosis of breast cancer. Oncotarget 2016;7:18485–94. 26918343 10.18632/oncotarget.7608 PMC4951303

[23] Klein EA , Richards D, Cohn A, Tummala M, Lapham R, Cosgrove D, . Clinical validation of a targeted methylation-based multi-cancer early detection test using an independent validation set. Ann Oncol 2021;32:1167–77. 34176681 10.1016/j.annonc.2021.05.806

[24] Shen SY , Singhania R, Fehringer G, Chakravarthy A, Roehrl MHA, Chadwick D, . Sensitive tumour detection and classification using plasma cell-free DNA methylomes. Nature 2018;563:579–83. 30429608 10.1038/s41586-018-0703-0

[25] Allouchery V , Perdrix A, Calbrix C, Berghian A, Lequesne J, Fontanilles M, . Circulating PIK3CA mutation detection at diagnosis in non-metastatic inflammatory breast cancer patients. Sci Rep 2021;11:24041. 34911971 10.1038/s41598-021-02643-y PMC8674263

[26] Christenson ES , Dalton WB, Chu D, Waters I, Cravero K, Zabransky DJ, . Single-nucleotide polymorphism leading to false allelic fraction by droplet digital PCR. Clin Chem 2017;63:1370–6. 28615231 10.1373/clinchem.2017.273177

[27] Chu D , Paoletti C, Gersch C, VanDenBerg DA, Zabransky DJ, Cochran RL, . ESR1 mutations in circulating plasma tumor DNA from metastatic breast cancer patients. Clin Cancer Res 2016;22:993–9. 26261103 10.1158/1078-0432.CCR-15-0943 PMC4993201

