# Supplementary material for: Development of an Automated Liquid Biopsy Assay for Methylated Markers in Advanced Breast Cancer
Source: Cancer Res Commun. 2022 Jun 1;2(6):391–401. doi: 10.1158/2767-9764.CRC-22-0133 (PMC9426415; doi:10.1158/2767-9764.CRC-22-0133)
Supplement: Supplementary Fig S1 — Figure shows the relationship between PCR Cycle threshold (Ct) and target DNA copy (0-300 copies) input [file crc-22-0133-s01.docx]

**Supplementary Fig. S1**

**Fig. S1 Relationship between PCR Cycle threshold (Ct) and target DNA copy input**. In this example, for ZNF671 the median ΔCt for ten replicates of 300 copies = 3.0 when methylated DNA was spiked into normal pooled plasma (Table S2). At 100% PCR reaction efficiency each 3.33 ΔCt increase is a 10-fold decrease in DNA copies, as indicated by the dotted lines. The heavy dashed threshold at ΔCts = 16 indicates the 3.0 Median value + 13 ΔCt units. A change in 13 Δ Ct units is approximately 2^13^- fold less than 300 copies in this example, or equal to 0.04 copies, a value below the limit of detection of LBx-BCM. Therefore, for the LBx-BCM methylation algorithm any individual gene methylation value higher than this dashed threshold is considered as a non-specific signal and the sample value adjusted to equal zero in step 2 of the LBx-BCM methylation algorithm (Table S1). No Amp: no signal seen during the run.
