# Supplementary material for: Development of an Automated Liquid Biopsy Assay for Methylated Markers in Advanced Breast Cancer
Source: Cancer Res Commun. 2022 Jun 1;2(6):391–401. doi: 10.1158/2767-9764.CRC-22-0133 (PMC9426415; doi:10.1158/2767-9764.CRC-22-0133)
Supplement: Supplementary Fig S2 — Figure shows the analytical sensitivity of LBx-BCM based detection of fully methylated DNA (0-300 copies) spiked into normal serum separately for each gene in the panel in 10-11 replicate assays [file crc-22-0133-s02.docx]

**Supplementary Fig. S2**

**Fig. S2 Analytical sensitivity of LBx-BCM.** Fully methylated DNA (300, 150, 75 and 0 copies) was spiked into 0.5 ml aliquots of pooled commercial normal serum in each of 10-11 replicate LBx-BCM assays. **A.** The ∆Ct (Ct Gene – Ct ACTB) of all test samples is plotted on the Y axis for each gene. The number of copies of methylated DNA spiked into serum is indicated on the X axis. For each gene, the median ∆Ct (Ct Gene – Ct ACTB) for 300 copies of spiked DNA is indicated numerically in red to the left of the Y-axis and by the lower dotted line. The upper dotted line indicates ∆Ct for 300 copies + 13 for each marker.  **B.** Cumulative methylation of the 9-gene panel for replicates. Mann-Whitney analysis shows significance (*P*-values) between groups of serum samples containing different numbers of spiked methylated DNA copies. The number of replicates performed and the coefficient of variation expressed as percent (CV %) is indicated.
