# Supplementary material for: Development of an Automated Liquid Biopsy Assay for Methylated Markers in Advanced Breast Cancer
Source: Cancer Res Commun. 2022 Jun 1;2(6):391–401. doi: 10.1158/2767-9764.CRC-22-0133 (PMC9426415; doi:10.1158/2767-9764.CRC-22-0133)
Supplement: Supplementary Fig S3 — Figure shows the perfomance of LBx-BCM in the training set samples. A histogram shows cumulative methylation for each sample, and a box plot shows significnt difference of methylation in serum of metastatic breast cancer patients compared to normal individuals (Mann Whitney p= 0.002) [file crc-22-0133-s03.docx]

**Supplementary Fig. S3**

**Fig. S3 Performance of LBx-BCM in the J0888 training set.** The LBx-BCM was performed on a set of 40 serum samples for the purposes of training the assay and the cumulative methylation (CM) algorithm (Methods and Table S1) on patient sera from the J0888 Repository. CM is plotted as a histogram, showing the extent of methylation (Y-axis) within individual samples (X-axis). The height of the colored segment of each bar indicates the relative amount of methylation present in individual genes. The dotted line indicates the ROC-derived threshold of CM = 38.5 units which provides the highest combined sensitivity and specificity. **B.** Shown in the box plot, the Mann-Whitney analysis indicates significantly (*P* = 0.002) higher methylation in metastatic breast cancer patients undergoing treatment compared to normal individuals.
