# Supplementary material for: Development of an Automated Liquid Biopsy Assay for Methylated Markers in Advanced Breast Cancer
Source: Cancer Res Commun. 2022 Jun 1;2(6):391–401. doi: 10.1158/2767-9764.CRC-22-0133 (PMC9426415; doi:10.1158/2767-9764.CRC-22-0133)
Supplement: Supplementary Fig S4 — Figure shows ACTB reference gene DNA levels plotted for each of the 132 study samples. Stage IV breast cancer patient sera had significantly higher total ACTB DNA (lower Ct) compared to normal (Mann Whitney P < 0.0001). Descriptive statistics are shown for normal vs. cancer and for methylation Cartridge A vs Cartridge B [file crc-22-0133-s04.docx]

**Supplementary Fig. S4**

**Fig. S4 ACTB levels in the LBx-BCM assay study samples**. **A**. Equal amounts of bisulfite treated DNA from 1 ml plasma was used to quantitate target gene methylation in each of two marker cartridges; Cartridge A: AKR1B1, TM6SF1, ZNF671, TMEFF2 normalized to ACTB reference; Cartridge B: COL6A2, HIST1H3C, RASGRF2, HOXB4, RASSF1 normalized to ACTB reference. All ACTB Ct levels were < 28.0 Ct. The difference in ACTB levels between cancer and normal plasma was statistically significant (Mann Whitney *P* < 0.0001. **B**. Descriptive statistics show that the endogenous reference gene ACTB Ct for Stage IV samples ranged from Ct 16.0 - 27.8, and for the normal samples ranged from Ct 21.0 - 27.4. Median and mean of ACTB Cts in the cancer and normal plasma, and in all samples (*N* = 132) are presented. Coefficient of variation expressed as percent (CV %) is indicated.
