# Supplementary material for: Development of an Automated Liquid Biopsy Assay for Methylated Markers in Advanced Breast Cancer
Source: Cancer Res Commun. 2022 Jun 1;2(6):391–401. doi: 10.1158/2767-9764.CRC-22-0133 (PMC9426415; doi:10.1158/2767-9764.CRC-22-0133)
Supplement: Supplementary Fig S5 — Figure shows changes in LBx-BCM methylation in response to chemotherapy in 7 additional patient longitudinal serial samples. LBx-BCM was performed and cumulative methylation (CM) (Y-axis) is plotted from serum samples drawn at baseline (0 days) and immediately before each treatment cycle (X-axis). For each patient, treatment cycles are shown as a shaded area. PD, progressive disease; SD, stable disease (SD). [file crc-22-0133-s05.docx]

**Supplementary Fig. S5**

**Fig S5. Changes in LBx-BCM methylation in response to chemotherapy.** In a continuation of Fig. 4, shown **(A-G)** are LBx-BCM analyses of seven additional longitudinal serial samples (J0214 and J0425 studies) of blood from patients undergoing treatment for MBC. LBx-BCM was performed and cumulative methylation (CM) (Y-axis) is plotted from serum samples drawn at baseline (0 days) and immediately before each treatment cycle (X-axis). For each patient, treatment cycles (21-28 days; C1, C2 etc.) are shown as a shaded area. PD, progressive disease; SD, stable disease (SD).
