# Supplementary material for: Development of an Automated Liquid Biopsy Assay for Methylated Markers in Advanced Breast Cancer
Source: Cancer Res Commun. 2022 Jun 1;2(6):391–401. doi: 10.1158/2767-9764.CRC-22-0133 (PMC9426415; doi:10.1158/2767-9764.CRC-22-0133)
Supplement: Supplementary Table S1 — Table contains sequences of primers and probes used to amplify the nine markers and ACTB in the LBx-BCM assay [file crc-22-0133-s06.docx]

**Table S1. Sequences of primers and probes used for amplification of gene panel in LBx-BCM assay**
