# Supplementary material for: Development of an Automated Liquid Biopsy Assay for Methylated Markers in Advanced Breast Cancer
Source: Cancer Res Commun. 2022 Jun 1;2(6):391–401. doi: 10.1158/2767-9764.CRC-22-0133 (PMC9426415; doi:10.1158/2767-9764.CRC-22-0133)
Supplement: Supplementary Table S2 — The table provides a step by step guide to calculate methylation in each gene followed by cumulative methylation using the algorithm derived in this paper. [file crc-22-0133-s07.docx]

**Table S2. Calculation of LBx-BCM Methylation, Algorithm Example***

*****Step 1: Obtain the Ct for each gene and the reference DNA from the GeneXpert® software. If no signal was detected assign Ct = 45. Calculate Δ Ct (Ct Gene – Ct ACTB) to normalize all genes to the reference (ACTB). Separately for each gene, transform all ΔCt to positive integers. If a negative ΔCt value is present for any sample, add a constant across all samples for that gene to return only positive integers (add that constant to the replicate control median ΔCt of 300 copies too). Step 2: Remove signals too low to quantitate. Change to Δ Ct = 0 if the Δ Ct is higher than the replicate control median ΔCt of 300 copies + 13 units (i.e, less than 0.04 copies of target DNA, Fig. S1). Step 3: Calculate the inverse of Δ Ct (Ct gene – Ct ACTB) so that high values have high methylation. Multiply by a factor of 1200 to increase the dynamic range of the data. This value now represents gene methylation (M). Step 4: Sum all M for the 9 genes in the marker panel to get the cumulative methylation (CM).
