# Supplementary material for: Development of an Automated Liquid Biopsy Assay for Methylated Markers in Advanced Breast Cancer
Source: Cancer Res Commun. 2022 Jun 1;2(6):391–401. doi: 10.1158/2767-9764.CRC-22-0133 (PMC9426415; doi:10.1158/2767-9764.CRC-22-0133)
Supplement: Supplementary Table S3 — The table provides descriptive statistics and coefficient of variation for methylation in each gene in replicate LBx-BCM analyses of 300 copies of fully methylated DNA spiked into normal serum or plasma [file crc-22-0133-s08.docx]

**Table S3. Analysis of technical replicates by the LBx-BCM assay**

*****Fully methylated DNA (300 copies) was spiked into 0.5 ml of serum or plasma.

**The LBx-BCM ΔCt of each replicate was calculated.

***The replicate median ΔCt = 300 copies of spiked DNA (bolded) for each gene was used to calculate cumulative methylation. Table supports data shown in Table S1, Fig. 1B and Fig. 2A.
